# Boredom Proneness and Self-Control as Unique Risk Factors in Achievement Settings

**DOI:** 10.3390/ijerph17239116

**Published:** 2020-12-06

**Authors:** Jhotisha Mugon, James Boylan, James Danckert

**Affiliations:** Department of Psychology, University of Waterloo, 200 University Avenue West, Waterloo, ON N2L 3G1, Canada; j2boylan@uwaterloo.ca (J.B.); jdancker@uwaterloo.ca (J.D.)

**Keywords:** boredom proneness, self-control, self-esteem, academic performance, GPA

## Abstract

The state of boredom arises when we have the desire to be engaged in goal pursuit, but for whatever reason we cannot fulfil that desire. Boredom proneness is characterized by both frequent and intense feelings of boredom and is an enduring individual difference trait associated with a raft of negative outcomes. There has been some work in educational settings, but relatively little is known about the consequences of boredom proneness for learning. Here we explored the unique contributions of boredom proneness, self-control and self-esteem to undergraduate self-reported higher grade point average (GPA). Within educational settings, prior research has shown self-control and self-esteem to be associated with better academic performance. In contrast, boredom proneness is associated with lower levels of self-control and self-esteem. Our analyses replicate those previous findings showing that self-control acts as a positive predictor of GPA. Importantly, we further demonstrated, for the first time, that boredom proneness has a unique contribution to GPA over and above the contribution of self-control, such that as boredom proneness increases, GPA decreases. We discuss potential mechanisms through which boredom proneness may influence academic performance.

## 1. Introduction

Boredom is a ubiquitous human experience in which individuals are motivated to engage with their environment but fail to successfully do so [1,2]. Although the bored individual is motivated to engage, they are not necessarily motivated to engage with their *immediate* environment. For example, a student may not be motivated to engage in their current learning environment, while being simultaneously highly motivated to engage in some *other* activity that they deem to be a more satisfying alternative. In that sense, the current environment can be seen as problematic since it is the impediment to successful engagement in alternatives. In other words, the state of boredom signals that what we are doing now is failing to satisfy our needs and goals in some important way, prompting us to explore alternative options for engagement. Previous research has demonstrated that monotony, lapses of attention, a lack of meaning, an unwillingness to engage in the task, subjective effort and agency are among the many situational variables that can lead to boredom [3,4,5,6,7,8,9,10,11,12].

Those who are high in boredom proneness then are individuals who, as a result of being consistently exposed to one or more of these antecedents, experience an increased frequency and intensity of state boredom [13]. That is, one recent paper suggests that boredom proneness is best characterized by the triumvirate of increased frequency and intensity of experiencing the state of boredom, together with a perceived lack of meaning (Tam et al., under consideration). This depiction of boredom is in line with previous research that has conceptualized state boredom as arising from a particular task [14,15]. However, it is noteworthy that this is not the only conceptualization of boredom. Feldges and Pieczenko (2020) differentiate between boredom directed *towards* an object (what they refer to as an emotion), from boredom that is non-intentional and is not directed toward any particular object (what they refer to as boredom as a mood; [16]). The notion that boredom can be in a sense “objectless” is an interesting one, perhaps best captured in a quote from Leo Tolstoy, that boredom is “a desire for desires” [17]. That is, we want something to engage with when bored but struggle to determine what that ought to be. This is in line with the functional account of state boredom—that state boredom signals rising opportunity cost and the need to explore one’s environs for some activity that is more engaging or rewarding than whatever is currently in front of us [7,18,19]. Regardless of whether boredom is conceptualized as a mood or an emotion [16], state boredom is consistently characterized as a negatively valenced experience [20] in the classic valence/arousal framework of describing emotions (for review see [21]). With regards to trait boredom it is well demonstrated in the literature that those high in trait boredom proneness experience a litany of negative outcomes such as increased depression and anxiety [22,23,24], susceptibility to problem gambling [25,26,27], increased absenteeism and decreased job satisfaction [28]. We have shown that boredom proneness is associated with lower levels of self-control, described as the ability to direct one’s thoughts and actions in the pursuit of goals—clearly a prerequisite for success in academic settings [29,30].

With respect to academic settings, research has also shown that state boredom is alarmingly prevalent. A recent study by Chin and colleagues (2016) used daily diary experience sampling to capture in-the-moment assessments of boredom coupled with information regarding what participants were doing at the time (e.g., school, gym, work, etc.) [31]. From a vast data sample (close to 4000 participants sampled multiple times over a 7–10 day period), their results showed that boredom was four times more prevalent in 25-year-olds than it was in 45-year-olds. More importantly, boredom was the fourth most common negative emotion reported and when it was reported it was three to six times more likely to occur while people were studying than doing other activities (e.g., playing sports, exercising). Furthermore, studying was associated with the highest rate of boredom compared to any other activities, even more so than when people reported doing nothing at all [31]. In a middle school setting, Daschmann and colleagues (2011) found that 44% of German students reported feeling bored in Mathematics classes [32]. Mann and Robinson (2009) asked university students in England to make retrospective judgements about how boring their lectures were, how much time during lectures they felt bored and whether having a boring lecture influenced their attendance to future lectures [33]. They found that 59% reported that at least half of their lectures were boring with 30% claiming that most or all of their lectures were boring. Furthermore, students who scored high on boredom proneness were more likely to rate time spent in lectures as boring and were more likely to skip future lectures compared to low boredom prone students [33]. These findings are consistent with findings of a meta-analysis by Tze and colleagues (2016) which found that between 26 and 41% of undergraduate students in Canada and 50% of undergraduates in China report being bored at some point during lectures [34].

Given the pervasiveness of state boredom in education settings, it is unsurprising that it has been related to poor academic outcomes. In a meta-analysis of 29 studies, Tze and colleagues (2016) found negative correlations between boredom and motivation to learn, use of learning strategies for deeper processing of information, and academic achievement (measured via test and assignment scores) [34]. The majority of the studies included in this analysis assessed state boredom, asking students about boredom experienced before/during and after classes. While a valid indication of the prevalence of state boredom in educational settings, it does not address any potential influence of trait boredom proneness on academic achievement.

Prior research has demonstrated that higher levels of self-control are associated with both lower levels of boredom proneness and greater academic success. Self-control may allow us to persist towards achievement of an academic goal in the face of various challenges, including feelings of boredom. Self-control has been defined as the ability to bring our thoughts, feelings and actions in line with our goals [35,36,37]. In multiple large samples of undergraduates, lower levels of self-control have been associated with higher levels of boredom proneness [29,30]. Within achievement settings, state boredom may pose two distinct challenges for learning, on the one hand making it harder to withstand distraction (i.e., sustaining attention on a task), while on the other making it difficult to select or focus on goals [38]. The relation between boredom, boredom proneness and poor performance on sustained attention tasks supports the notion that the kind of focused attention necessary for learning is compromised both when we are bored in the moment and by the trait propensity for experiencing the state [39]. With regards to self-control, Tangney and colleagues (2004) found that undergraduate students who reported higher levels of self-control had higher grade point averages (GPA) and higher self-esteem compared to those with low self-control [37]. Similarly, Duckworth and colleagues (2010) followed a group of 5th grade students for 4 years and found that self-control was positively associated with both self-esteem and GPA, while self-esteem alone was marginally associated with GPA [40]; see also [41,42] for mixed results on academic achievement and self-esteem.

Given the opposing effects of self-control and state boredom on achievement, we explored whether trait boredom proneness was also associated with academic achievement above and beyond trait self-control. We hypothesized that within our undergraduate sample, low self-control and high boredom proneness would both be associated negatively with GPA (i.e., low self-control and high boredom proneness would be correlated with poor academic achievement). Of note here are the potential effects of self-esteem on GPA (mentioned above) and the role of age on self-control. Despite the small age variation in typical undergraduate samples, age has been found to correlate positively with self-control, likely because such age groups experience some of the most rapid development in frontal-lobe maturation, which in turn is correlated with increases in self-control [29,43]. Consequently, Isacescu and colleagues (2017) also observed a small yet significant negative correlation between age and boredom proneness even when the age range tested was restricted to 17- to 22-year-olds [29]. Given that age and self-esteem have both been shown to correlate with self-control and better performance in academic settings, we included both of these variables in our analysis to enable us to determine the unique contribution of boredom proneness to GPA over and above these factors [37,40]. To explore the unique contributions of each trait-level construct on GPA, we conducted both regression analyses and mixed effects models with boredom proneness, self-control and self-esteem predicting GPA. These analyses allowed us to determine the amount of variance in GPA uniquely accounted for by boredom proneness when self-control, age and self-esteem were also taken into consideration.

## 2. Materials and Methods

### 2.1. Participants

We used a convenience sample of data collected from undergraduate participants across seven semesters—each at the beginning of Fall 2016, 2017, 2018, Spring 2017, 2018 and Winter 2017, 2018, as part of an online survey routinely administered to undergraduates at the University of Waterloo. Undergraduate students participate in these online surveys for course credit. Our overall sample consisted of 8182 unique participants—many of whom completed the survey in multiple terms (see Table 1 for the breakdown for each term). All participants gave informed consent prior to completing the questionnaires and the study was approved by the University of Waterloo’s Office of Research Ethics (approval code: 40884).

### 2.2. Self-Report Measures

Boredom Proneness: The Shortened Boredom Proneness Scale (SBPS) is an 8-item questionnaire designed to assessed one’s proneness to experience boredom. Sample items include “I find it hard to entertain myself” measured on a Likert scale ranging from 1 “strongly disagree” to 7 “strongly agree” with high scores reflecting a high propensity to be bored [44]. Struk and colleagues (2017) report an internal consistency of 0.88 [44]. We chose to use this scale instead of the original 28-item boredom proneness scale [45] based on a study that compared the original scale with the shortened version and found that the shortened version better captured trait boredom proneness [46].

Self-Control: Trait self-control was measured using the Brief Self-Control Scale (BSCS) which is a 13-item scale that assesses trait self-control one has over one’s cognitions, emotions and behaviors. Sample items include “I am good at resisting temptation” measured on a 5-point Likert scale ranging from 1 “not at all” to 5 “very much” with high scores reflecting high self-control [37]. Tangney and colleagues (2004) report an internal consistency of 0.83 and 0.85 across two studies and a test–retest reliability of 0.87 [37].

Self-Esteem: To measure self-esteem we used the Rosenberg Self-Esteem Scale (RSES), which is a 10-item scale including items such as “On the whole, I am satisfied with myself.” In this study, items were measured on a 9-point Likert scale ranging from 1 “very strongly disagree” to 9 “very strongly agree” with high scores reflecting high self-esteem [47]. Sinclair and colleagues (2010), report internal consistency ranging from 0.57–0.79 and a test–retest reliability of 0.91 from a large representative sample [48].

Grade Point Average (GPA): GPA was measured using one question in which participants were asked to select the category that best represents their current grades. GPA was binned into 5-point ranges (e.g., 60–64.9%, 65–69.9%, 70–74.9%, etc.). The only exception to these categories was for participants who had a GPA of less than 60%, at which point they selected the category <60%. During data analyses, each of these categories were converted into a numeric scale ranging from 1 (<60%) to 9 (95–100%). The GPA question formed part of a pre-screen questionnaire administered to participants at the beginning of term as part of the online survey package. Therefore, when reporting their GPA, participants’ scores are representative of their current GPA (hereafter referred to as Term 0 or T0: that is T0 refers to the first, baseline point of GPA measurement). Although participants had to rely on their memory to report their GPA, they would have received their final official GPA within 2–3 weeks at the time of reporting. This represents a relatively small time difference that would be expected given the design and administration of the mass testing scales. Although we cannot fully ascertain the accuracy of this measure, we observed a high test–retest reliability of 0.71 among our samples. Furthermore, students knew that their responses were anonymous and thus had little incentive to lie about their actual GPA. Any inaccuracies in self-reporting, as is true with any other self-report measure, are likely to introduce noise and thus may attenuate the strength of observed relationships between GPA and our variables.

## 3. Results

All statistical analyses were conducted using R statistical software [49]. Descriptive statistics for our variable of interests for each term are presented in Table 2. There were high levels of test–retest consistency for all variables across the seven terms. For example, pairwise correlations using the same individuals who contributed to Fall 2016 (Time 1) and Fall 2018 (Time 7) showed a significant relationship between boredom proneness scores at Time 1 and Time 7 (*r*(311) = 0.56, *p* < 0.0001). Table 3 shows pairwise correlations between the first term collected (F16) and each subsequent term for each measure of interest (note, in each correlation only individuals who completed surveys in both terms are included, hence the fluctuating sample sizes).

Tests of normality indicated that all variables across all terms were not normally distributed. Therefore, to test whether there was a relationship between SBPS and GPA, we conducted Spearman correlations for each term. In an attempt to replicate previous findings, we also report the relations between GPA and BSCS and RSES in Table 4. There were no significant differences in grades between males and females (all *p*-values > 0.05). With regards to trait measures, Wilcoxon rank sum tests revealed that males reported significantly higher levels of boredom proneness than females in W17 and Spring 2018 (*p*-values < 0.01 for both instances). Males reported higher levels of self-esteem than females in F17, W18 and F18 (all *p*-values < 0.008). Females reported higher levels of self-control than males in all terms with the exception of S17 and S18 (all *p*-values < 0.04). With regards to age, Spearman correlations suggested a negative relationship between boredom proneness and age for F16 and F18 (all *p*-values < 0.02), a positive relationship between self-esteem and age for F16, F17 and F18 (all *p*-values < 0.04), and no significant relationship between self-control and age (all *p*-values > 0.5). Across all terms, boredom proneness was significantly negatively correlated with self-control (*r* = −0.46 to −0.53), self-esteem (*r* = −0.54 to −0.58) and GPA (with the exception of Spring 2017; *r* = −0.13 to −0.23). Self-control and self-esteem were positively correlated (*r* = 0.39 to 0.51). GPA was positively associated with self-control (*r* = 0.19 to 0.27) and self-esteem (except for spring 2017; *r* = 0.1 to 0.13).

Regression models were calculated for each term to determine whether each of our trait measures coupled with age and gender were uniquely associated with GPA. Self-control was consistently positively and uniquely associated with grades across all seven terms, while boredom proneness was negatively and uniquely associated with grades for the F16, F17 and S18 terms. Furthermore, age was negatively and uniquely associated with grades for F16, W18 and F18, while there were no significant associations between gender and grade. Self-esteem showed no relation to grades in any term (Table 5).

The mixed results from the regressions may be due to the fact that we are dealing with relatively small effect sizes. To address this, we opted to construct a single powerful test in the form of a linear mixed effects model by combining observations across all terms. If our variables of interests truly have unique effects on GPA, it should be evident in such a model. Since collapsing across terms would mean that we have multiple measures from some participants (i.e., those participants who completed the same questionnaires in more than one term), participant was set as the random effects variable and boredom proneness, self-control, self-esteem, age and gender were set as fixed effects. The mixed effects model was calculated using the lme4 package [50] and *p*-values were obtained with the lmerTest package [51]. The model was fitted using restricted maximum likelihood (REML) and *t*-tests were calculated using the Satterthwaite’s method. Within this model, we had 5770 unique participants with the total number of observations being 7925. Table 6 shows the fixed effects from the linear mixed model. The results suggest that both boredom proneness and self-control make unique contributions to GPA in that boredom proneness was negatively associated with GPA, while self-control was positively associated with GPA. Age, gender and self-esteem were not significantly associated with grades. We also calculated fixed effects from another linear mixed effects model with our variables of interest predicting GPA collected in the following term (i.e., the independent variables are lagged by a term). Results from this model were the same as for the original model (i.e., when GPA and IVs were collected simultaneously). The exception was with regard to the age variable which in the new model was a significant correlate of GPA (Age: Estimate = 0.05, SE = 0.02, df = 1535, *t* = 3.57, *p* < 0.001; Gender: Estimate = −0.08, SE = 0.10, df = 1260, *t* = 0.87, *p* = 0.383; SBPS: Estimate = −0.10, SE = 0.04, df = 1198, *t* = 2.72, *p* = 0.007; RSES: Estimate = 0.04, SE = 0.03, df = 1420, *t* = 1.48, *p* = 0.139; BSCS: Estimate = 0.31, SE = 0.07, df = 1455, *t* = 5.262, *p* < 0.001). Since we had two significant correlates of GPA we opted to conduct a further analysis to determine whether there were any significant interactions between boredom proneness and self-control in predicting GPA. To this effect, an interaction term between self-control and boredom proneness was entered in the linear mixed effects model, with results suggesting that the interaction term was not significant (Estimate = 0.0375 S.E. = 0.02538, *t* = 1.48, *p* = 0.139).

## 4. Discussion

Our findings suggest that trait boredom proneness is uniquely associated with GPA, above and beyond the contribution of self-control. Clearly, trait levels of self-control were the strongest predictor of GPA. While boredom proneness is not as prominent a contributor to GPA, it does appear to have a unique contribution which future work should explore further. That said, the strength of the correlations between boredom and GPA are on par with other commonly used predictors of academic achievement. For example, subscales of the Motivated Strategies for Learning Questionnaire (MSLQ), a tool widely used to predict academic achievement [52], show similar strengths of correlation between the subscales and GPA (see [53] for a meta-analytic review of the MSLQ and its association with GPA). Given that boredom proneness is identified as a unique risk factor, it may represent a novel vector for intervention or theoretical inquiry.

As in previous work, age and gender showed expected (although somewhat inconsistent) effects on boredom proneness (i.e., with boredom proneness diminishing with age and being slightly higher in males; see also [29,30]). Interestingly, self-esteem was not a reliable predictor of GPA in our sample. As mentioned in the introduction, the effect of self-esteem on GPA has been inconsistent in the literature [40,41,42]. In the current study, we found that self-esteem was strongly, negatively correlated with boredom proneness and positively associated with self-control, but also that self-esteem did not uniquely contribute to prediction of academic performance. Indeed, Baumeister and colleagues (2003), using path analyses, argued that self-esteem should be considered a consequence rather than a cause of positive academic achievement [54]. One reason why self-esteem may not uniquely contribute to academic performance is that the Rosenberg Self-Esteem Scale (1965) measures our global representation of our sense of self-worth. Students may consider academic achievement to be irrelevant, or at least only partly contributing, to appraisals of their overall self-worth [47,54]. Previous research has found that using a more specific measure of self-worth, such as academic self-concept, does successfully predict grades [55]. Nonetheless, the observed small positive correlations between GPA and self-esteem are consistent with previous findings [56,57].

While the current work suggests that boredom proneness is uniquely predictive of lower GPA, we are still left with the question of how this relation arises. Research suggests that in-the-moment feelings of boredom are most prominent when students feel over- or under-challenged [58,59]. As such, those high in boredom proneness may be seeking a kind of “Goldilocks” zone of skill-challenge fit to promote optimal engagement, something they struggle to achieve. Any failure to find an optimally engaging circumstance in turn should have detrimental consequences for how well the student engages with the material or sustains attention in class. We know that boredom, as both a state and trait, is predictive of poor performance on sustained attention tasks [39,60], which would clearly be detrimental to learning [61,62,63]. For example, Mann and Robinson (2009) found that the use of PowerPoint slides is likely to elicit boredom among students and that boredom prone students who report experiencing more boredom during lectures and are also more likely to miss lectures [33]. In other words, if state boredom signals that we are disengaged, and the highly trait boredom prone experience this state more frequently and intensely, then the clearest possible explanation for our results is that boredom proneness has a negative impact on GPA by making it difficult for the student to engage with the material at hand. The direction of causality is of course speculative on our part and will require further investigation.

In a related sense, boredom proneness has been characterized by us as a failure to launch into action [1]. That is, boredom proneness represents a kind of conundrum—the boredom prone individual recognizes the regulatory signal that is state boredom as a call to action, but they fail to adaptively respond to it [64]. For the highly boredom prone, for whatever reason, the call to action is either maladaptively responded to (e.g., increased risk taking, impulsivity, a variety of addictions) [25,26,60,65,66,67], or is not responded to at all (i.e., boredom prone individuals tend to procrastinate) [68]. In the context of education settings then, boredom proneness may represent a barrier to effective engagement with the material. Indeed, research by Nett and colleagues (2010) demonstrated that students’ boredom coping strategies tend to fall on two dimensions—a cognitive vs. behavioral dimension and an approach vs. avoidance dimension [69]. The authors found that those who used cognitive-approach strategies such as re-appraising the value of the situation when bored in class, were more effective in dealing with the state compared to those who employed avoidance strategies. In a follow up paper, the authors also found that those who employ cognitive approach strategies are less likely to be boredom prone and that boredom prone students are more likely to employ avoidance strategies such as behaviors that disrupt the lesson or working on other subjects [70].

One limitation of this study is that our grade measure was categorical. This meant that our measure may not have been sensitive to more granular changes in GPA (i.e., a student experiencing an increase from 75% to 79% would have to select the same GPA category). Future studies should employ a more fine-grained metric of academic performance to more closely examine the influence of boredom proneness on academic achievement. Even so, given that boredom proneness was negatively associated with grades in our current sample, we predict that with the use of more granular measures of GPA, the strength of this relationship should increase.

While our results highlight the relationship between boredom proneness and GPA, they do not tell us anything about the direction of causality. That is, does boredom proneness cause a decrement in GPA or do achievement failures cause students to become more boredom prone? Existing research findings hint at a possible causal direction: that self-control may be an antecedent to both positive and negative academic emotions (defined here as emotions experienced during achievement related settings) including boredom [71]. Using path analyses, King and colleagues (2014) found that individuals with low self-control were more likely to experience negative emotions (such as boredom, anger, anxiety) in their university English class, which in turn led to passive attitudes or withdrawn engagement from learning activities and which consequently had negative consequences for GPA [71]. While the researchers did not specifically differentiate boredom from the other negative emotions, this finding is in line with our notion that boredom proneness is prominently associated with a failure to launch into action or to engage effectively with a task at hand. Future research longitudinal research should explore whether boredom proneness causes individuals to disengage over time and whether this in turn is associated with lower academic performance.

The current findings have important implications for educators. Best practices to maintain engagement in the classroom range from creating interesting content, raising learner curiosity and applying active learning strategies [72,73,74,75]. While these practices may keep the experience of boredom at bay, the negative relationship between boredom proneness and self-control highlights the need for instructors to foster self-control strategies in their learners. One key factor not tested here is likely to play a role in both self-control and boredom proneness in academic settings—one’s sense of agency [38]. That is, when learners feel empowered by the belief that their own actions and learning strategies have a high chance of breeding successful engagement and achievement, feelings of boredom are more likely to be kept at bay.

## 5. Conclusions

This study explored the unique contributions of boredom proneness, self-esteem, and self-control to GPA within a university convenience sample. Across seven terms, there were high levels of test–retest consistency for all variables. Across all terms, boredom proneness was significantly negatively correlated with self-control, self-esteem, and GPA (except for Spring 2017). Self-control and self-esteem were positively correlated with one another and GPA was positively associated with self-control and self-esteem (except for spring 2017).

Regression models were calculated for each term to determine the unique contributions of each variable to GPA. Self-control was consistently positively and uniquely associated with grades across all seven terms, while boredom proneness was negatively and uniquely associated with grades for the F16, F17 and S18 terms. Self-esteem showed no relation to grades in any term. Due to mixed results from the regressions, a more powerful linear mixed effects model was used to test the unique effects of each variable on GPA. Participant was set as the random effects variable and boredom proneness, self-control, self-esteem, age and gender were set as fixed effects. Results suggest that both boredom proneness and self-control make unique contributions to GPA in that boredom proneness was negatively associated with GPA, while self-control was positively associated with GPA. No other significant associations were observed. While boredom proneness is not as big of a contributor to GPA as self-control is, the fact that it is uniquely associated with GPA warrants future investigation into the direction of this relationship.

## Figures and Tables

**Table 1:** Participant Breakdown by Term.

Term	# of Participants (Females)	Mean Age in Years (SD):
Fall 2016	2660 (1902)	21.28 (2.7)
Winter 2017	2232 (1599)	21.41 (2.2)
Spring 2017	205 (153)	22 (3.6)
Fall 2017	2195 (1630)	22 (2.9)
Winter 2018	2105 (1523)	20.58 (3.1)
Spring 2018	494 (328)	21.54 (3.5)
Fall 2018	2184 (1602)	20.4 (3.1)

**Table 2:** Descriptive Statistics.

Term	N	Grade Mean (SD)	SBPS Mean (SD)	BSCS Mean (SD)	RSES Mean (SD)
1. F16	2660	5.59(1.52)	3.26 (1.17)	2.96 (0.67)	5.96 (1.58)
2. W17	2232	5.31 (1.51)	3.27 (1.17)	2.97 (0.66)	5.98 (1.6)
3. S17	205	5.45 (1.54)	3.24 (1.16)	2.64 (0.67)	5.71 (1.6)
4. F17	2195	5.76 (1.56)	3.3 (1.14)	2.9 (0.67)	5.85 (1.62)
5. W18	2105	5.55 (1.48)	3.26 (1.17)	2.63 (0.66)	5.89 (1.62)
6. S18	494	5.26 (1.58)	3.4 (1.2)	2.57 (0.7)	5.79 (1.57)
7. F18	2184	5.95 (1.53)	3.33 (1.67)	2.64 (0.66)	5.77 (1.63)

Term: F = Fall, W = Winter, S = Spring; SBPS = Short Boredom Proneness Scale; BSCS = Brief Self-control scale; RSES = Rosenberg Self-Esteem Scale.

**Table 3:** Longitudinal Consistency across Measures ^1^.

Measure	Term 1 × 2	Term 1 × 3	Term 1 × 4	Term 1 × 5	Term 1 × 6	Term 1 × 7
SBPS	*r*(839) = 0.71	*r*(59) = 0.65	*r*(606) = 0.66	*r*(473) = 0.67	*r*(109) = 0.66	*r*(311) = 0.56
BSCS	*r*(829) = 0.78	*r*(55) = 0.83	*r*(594) = 0.73	*r*(463) = 0.72	*r*(107) = 0.67	*r*(305) = 0.61
RSES	*r*(826) = 0.79	*r*(56) = 0.81	*r*(595) = 0.76	*r*(460) = 0.74	*r*(107) = 0.74	*r*(302) = 0.66
GPA	*r*(708) = 0.7	*r*(50) = 0.79	*r*(496) = 0.60	*r*(395) = 0.62	*r*(84) = 0.57	*r*(251) = 0.48

All correlations significant at *p* < 0.0001. ^1^ Note that these are pairwise correlations and the n diminishes for each subsequent correlation for a few reasons: (1) only students who take psychology courses are eligible for such questionnaires—some students may not take psychology courses two terms in a row; (2) students who are enrolled in the co-operative program alternate between a work term and a study term, and (3) regular full time students normally do not take classes in Spring terms, hence the lower sample sizes in these terms.

**Table 4:** Cross Sectional Correlations by Term.

Term	SBPS × BSCS	SBPS × RSES	BSCS × RSES	SBPS × GPA	BSCS × GPA	RSES × GPA
1. F16	*r*(2610) = −0.47 ***	*r*(2609) = −0.58 ***	*r*(2600) = 0.43 ***	*r*(2244) = −0.16 ***	*r*(2221) = 0.19 ***	*r*(2220) = 0.13 ***
2. W17	*r*(2193) = −0.46 ***	*r*(2191) = −0.56 ***	*r*(2184) = 0.41 ***	*r*(2031) = −0.13 ***	*r*(2019) = 0.19 ***	*r*(2016) = 0.12 ***
3. S17	*r*(202) = 0.53 ***	*r*(201) = −0.57 ***	*r*(201) = 0.51 ***	*r*(183) = −0.11 ^n.s^	*r*(180) = 0.27 **	*r*(180) = 0.1 ^n.s^
4. F17	*r*(2147) = −0.49 ***	*r*(2141) = −0.58 ***	*r*(2136) = 0.41 ***	*r*(1825) = −0.17 ***	*r*(1808) = 0.23 ***	*r*(1804) = 0.13 ***
5. W18	*r*(2073) = −0.48 ***	*r*(2067) = −0.58 ***	*r*(2062) = 0.43 ***	*r*(1937) = −0.13 ***	*r*(1923) = 0.20 ***	*r*(1918) = 0.13 ***
6. S18	*r*(492) = −0.52 ***	*r*(491) = −0.54 ***	*r*(490) = 0.43 ***	*r*(429) = −0.23 ***	*r*(428) = 0.21 ***	*r*(427) = 0.10 *
7. F18	*r*(2155) = −0.5 ***	*r*(2151) = −0.57 ***	*r*(2138) = 0.39 ***	*r*(1821) = −0.13 ***	*r*(1800) = 0.20 ***	*r*(1802) = 0.13 ***

*** = *p* < 0.0001, ** = *p* < 0.001, * = *p* < 0.05, n.s = not significant.

**Table 5:** Regression Models Looking at the Unique Contributions of Each Variable to grade point average (GPA).

Term	Regression Model		Age		SBPS		RSES			BSCS		
F16	*F*(5, 2164) = 22.81 ***	*R^2^* = 0.05	*β* = −0.07	*t* = −3.5 ***	*β* = −0.09	*t* = −3.5 ***		*β* = 0.01	*t* = 0.29		*β* = 0.15	*t* = 6.01 ***
W17	*F*(5, 1946) = 18.34 ***	*R^2^* = 0.05	*β* = 0.03	*t* = 1.36 ^n.s^	*β* = −0.04	*t* = −1.5 ^n.s^		*β* = 0.01	*t* = 0.49 ^n.s^		*β* = 0.18	*t* = 6.9 ***
S17	*F*(5, 168) = 2.37 *	*R^2^* = 0.04	*β* = 0.05	*t* = 0.65 ^n.s^	*β* = 0.04	*t* = 0.39 ^n.s^		*β* = −0.02	*t* = −0.25 ^n.s^		*β* = 0.27	*t*= 2.96 **
F17	*F*(5, 1752) = 21.54 ***	*R^2^* = 0.06	*β* = −0.006	*t* = −0.25 ^n.s^	*β* = −0.06	*t* = −2.16 *		*β* = 0.03	*t* = 0.95 ^n.s^		*β* = 0.19	*t* = 6.77 ***
W18	*F*(5, 1865) = 18.97 ***	*R^2^* = 0.05	*β* = −0.05	*t* = −2.24 *	*β* = −0.03	*t* = −0.85 ^n.s^		*β* = 0.04	*t* = 1.27 ^n.s^		*β* = 0.18	*t* = 6.89 ***
S18	*F*(5, 401) = 6.71 ***	*R^2^* = 0.07	*β* = 0.03	*t* = 0.53 ^n.s^	*β* = −0.21	*t* = −3.39 ***		*β* = −0.06	*t* = −1.09 ^n.s^		*β* = 0.13	*t* = 2.27 *
F18	*F*(5, 1744) = 34.61 ***	*R^2^* = 0.09	*β* = −0.21	*t* = −9.34 ***	*β* = −0.03	*t* = −0.97 ^n.s^		*β* = 0.05	*t* = 1.89 ^n.s^		*β* = 0.18	*t* = 6.59 ***

*** *p* < 0.001, ** *p* < 0.01, * *p* < 0.05; Gender was included in the models but is not reported here as it was non-significant in all terms.

**Table 6:** Fixed effects from linear mixed effects model predicting GPA.

	Estimate	Std. Error	95% CI	df	*t*-Value
(Intercept)	4.76	0.21	(4.35, 5.16)	7781	22.89 ***
Age	−0.01	0.01	(−0.02, 0.01)	6681	−1.07
Gender	0.02	0.04	(−0.06, 0.11)	5807	0.56
SBPS	−0.07	0.02	(−0.10, −0.04)	7309	−3.97 ***
RSES	0.02	0.01	(0.00, 0.05)	7808	1.74
BSCS	0.35	0.03	(0.30, 0.41)	7794	12.56 ***

*** *p* < 0.001.
